# Cytogenetic and Molecular Predictors of Outcome in Acute Lymphocytic Leukemia: Recent Developments

**DOI:** 10.1007/s11899-012-0122-5

**Published:** 2012-04-20

**Authors:** Ilaria Iacobucci, Cristina Papayannidis, Annalisa Lonetti, Anna Ferrari, Michele Baccarani, Giovanni Martinelli

**Affiliations:** 1 Department of Hematology and Oncological Sciences “L. and A. Seràgnoli”, University of Bologna, Via Massarenti, 9, 40138 Bologna, Italy; 2 Department of Human Anatomy, University of Bologna, Cellular Signalling Laboratory, Bologna, Italy

**Keywords:** ALL, Acute lymphoblastic leukemia, SNP array, Next generation sequencing

## Abstract

During the last decade a tremendous technologic progress based on genome-wide profiling of genetic aberrations, structural DNA alterations, and sequence variations has allowed a better understanding of the molecular basis of pediatric and adult B/T- acute lymphoblastic leukemia (ALL), contributing to a better recognition of the biological heterogeneity of ALL and to a more precise definition of risk factors. Importantly, these advances identified novel potential targets for therapeutic intervention. This review will be focused on the cytogenetic/molecular advances in pediatric and adult ALL based on recently published articles.

## Introduction

**Fig. 1:**
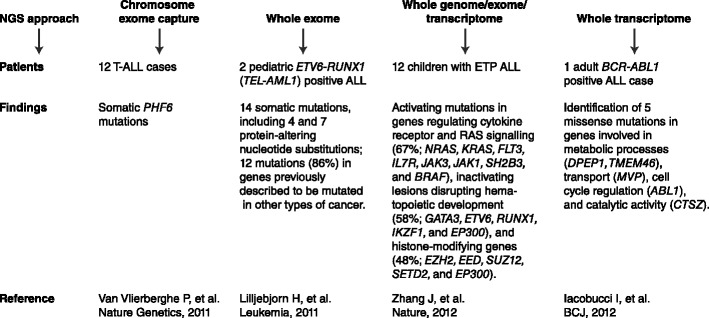
Next-generation sequencing (NGS) in B/T ALL. Whole genome/exome/transcriptome sequencing approaches provide a comprehensive view of the landscape of genetic alterations in leukemia allowing the identification of potential novel markers for diagnosis, risk-stratification, and tailored treatments. Here, studies using NGS approaches in B/T ALL are schematized

ALL represents a biologically and clinically heterogeneous group of B/T-precursor-stage lymphoid cell malignancies arising from genetic insults that block lymphoid differentiation and drive aberrant cell proliferation and survival. Incidence and cure rates differ among children and adults. In children, ALL is the commonest malignancy accounting for approximately 25 % of childhood cancer and it has 5-year event-free survival rates ranging between 76 % and 86 % in patients receiving protocol-based therapy. In adults, ALL is less common and generally carries a worse prognosis with a long-term survival probability less than 35–40 % [1, 2]. Although there is remarkable progress made in the treatment of ALL in children and, with less efficacy, in adults, several ALL subtypes continue to have a poor prognosis and in a proportion of long-term surviving patients, treatment is responsible for short and long-term toxicities. Consequently, there is a need in improving the molecular dissection of subtypes, identifying genetic alterations that predict the risk of treatment failure, and developing novel and targeted therapies. Cytogenetics has long been used for diagnosis, risk stratification, and therapeutic implications, however experimental models [3, 4] have established that primary cytogenetic abnormalities alone are insufficient to induce leukemia and that cooperative mutations are required. The development of microarray technologies to profile gene expression and structural genetic alterations in a genome-wide and high-resolution fashion have revolutionized our ability to identify genetic abnormalities providing important insights into the pathways deregulated in ALL [5]. Moreover, recently the development of next-generation sequencing (NGS) technologies has provided researchers with completely new and effective tools for the discovery of novel alterations, depicting an exhaustive picture of the leukemia genome complexity (Fig. 1). This review emphasizes the most important and newest findings obtained from genomic analysis of B/T-lineage ALL.

## B-Progenitor Acute Lymphoblastic Leukemia

### Prognostic Factors Based on Cytogenetics

Cytogenetics and fluorescence in situ hybridization (FISH) reveal recurring chromosome abnormalities, including numerical and structural changes, in approximately 80 % of ALL. Substantial differences in the frequencies of some recurring abnormalities are present between children and adults.

- The t(8;14)[*MYC-IgH*], present in a high proportion of Burkitt cell leukemia/lymphoma, occurs in approximately 1 % of adults. This translocation is associated with French-American-British (FAB)-L3-type leukemia cells, high incidence of central nervous system involvement at diagnosis, and poor prognosis [6].
- The t(4;11)[*MLL-AF4*] is present in up to 60 % of infants younger than 12 months, but is rarely observed in adult patients. This translocation is associated with FAB L1 or L2 morphology, immature immunophenotype, B-cell lineage, frequent co-expression of myeloid antigens, and high leukocyte counts. The prognosis is poor in both adults and children [7, 8].
- The t(9;22)[*BCR-ABL1*] that produces the Philadelphia (Ph) chromosome is observed in about 2 % to 5 % of children and 30 % of adults. This translocation is associated with a dismal prognosis. Treatment with tyrosine kinase inhibitors (TKIs) has produced promising results, although with the passage of time, problems have emerged with drug resistance and disease persistence [9].
- The t(1;19)[*TCF3-PBX1*] occurs in approximately 30 % of pre-B cell childhood ALL patients. In the past, patients with the t(1;19) typically had early treatment failure. However, this adverse prognosis can be overcome by more intensive chemotherapy such that the t(1;19) is now associated with a favorable prognosis [10, 11].
- The t(12;21)[*ETV6-RUNX1*] is detectable by FISH or polymerase chain reaction analysis in about 25 % of children and 3 % of adults with B-ALL. Patients generally have a favorable prognosis [12].
- Patients with hyperdiploidy with more than 50 chromosomes often have a good prognosis. The individual structural abnormalities do not appear to influence outcome in patients with hyperdiploidy except for the t(9;22), which is associated with a poor prognosis [13].
- Approximately 5 % to 6 % of ALL patients, independent of age, have clonal loss of various chromosomes, resulting in a hypodiploid clone with fewer than 46 chromosomes. These patients generally have a poor prognosis, especially those with near-haploid and low-hypodiploid clones. Similarly, deletion of 9p is an unfavorable risk factor associated with a high rate of relapse in B-precursor ALL in children [14].

### Prognostic Factors Based on Genomic Profiling

The advent of high-resolution genome-wide analyses of gene expression, DNA copy number alterations (CNA), and loss of heterozygosity have led to the detection of many novel genetic abnormalities refining the prognostic models for ALL (Table 1). Using single nucleotide polymorphism (SNP) arrays, many groups detected multiple submicroscopic CNA not evident on cytogenetic analysis, commonly less than one megabase (Mb) in size and targeting, in most of the cases, a single or few genes implicated in key cellular pathways, such as lymphoid development (*IKZF1, PAX5, EBF1, VPREB1*), cell cycle regulation and tumor suppression (*CDKN2A/CDKN2B, PTEN, BTG1, RB1*), lymphoid signaling (*BTLA, CD200*), drug responsiveness (*NR3C1*), and DNA mismatch repair (*MTOR, HERC1, PRKCZ,* and *PIK3C2B*) [5, 15–17, 18•, 19, 20]. Importantly, there were substantial differences in the frequency and nature of CNAs among various disease subtypes. *MLL*-rearranged leukemias harbor less than one CNA per case, suggesting that *MLL* is a potent oncogene that requires very few cooperating lesions to induce leukemia transformation. In contrast, *ETV6-RUNX1* and *BCR-ABL1* rearranged leukemias harbor six to eight alterations per case that may be present years after the initial chromosome rearrangements, indicating that these additional postnatal genetic alterations are required to induce the full leukemic phenotype [5].

**Table 1:** Novel recurring genetic alterations occurring in B-progenitor ALL and their correlation with outcome

Gene name	Genetic alteration	Frequency	Prognosis	References
*IKZF1*	Deletions or sequence mutations	~80 % of *BCR-ABL1* positive ALL; 15 % of pediatric B-ALL cases	Associated with poor outcome	[5, 27–31]
*PAX5*	Deletions, sequence mutations or translocations	~30 % of pediatric and adult B*-*ALL	No association with outcome	[17, 29, 35]
*CDKN2A/B*	Deletions	~30 % of pediatric and adult B*-*ALL; 47 % of relapsed *BCR-ABL1*-ALL	Associated with poor outcome in adult *BCR-ABL1* positive ALL; controversial prognosis in other B-ALL subtypes	[18•, 87, 88]
*CRLF2*	Overexpression due to *IGH@-CRLF2* or *P2RY8-CRLF2* rearrangement or F232C mutation	Up to 16 % of pediatric and adult B*-*ALL; >50 % Down syndrome ALL	Associated with poor outcome	[40–43]
*JAK1/2*	Sequence mutations	~10 % of high-risk *BCR-ABL1*-like ALL and 18–35 % of Down syndrome ALL	Associated with poor outcome	[40–43]
*CREBBP*	Deletions and sequence mutation	19 % of relapsed B-ALL	Associated with glucocorticoid resistance	[54•]
iAMP21	Intrachromosomal amplification	Up to 2 % in older children with B- ALL	Associated with poor outcome when patients are treated with standard therapy	[50, 51]
*TP53*	Deletions and sequence mutations	12.4 % of B-ALL	Associated with non-response to chemotherapy and poor event-free survival and overall survival rates	[56]

#### Genetic Alterations in B-Lymphoid Development Genes

Over two-thirds of genetic abnormalities affected genes involved in lymphoid development. These alterations include deletions, focal amplifications, novel translocations, and point mutations involving transcriptional regulators of early B-cell lineage development, such as *IKZF1* (Ikaros), *PAX5* (Paired box 5), *EBF1* (early B-cell factor), *LEF1* (lymphoid enhancer factor 1), and immunoglobulin family genes, such as *VPREB1* (pre-B lymphocyte 1).

##### IKZF1

Ikaros is a DNA-binding zinc finger transcription factor that regulates the development and function of the immune system and acts as a master regulator of normal hematopoietic differentiation and proliferation, particularly in lymphoid lineages [21]. During the last 5 years, *IKZF1* has been established as one of the most clinically relevant tumor suppressors in ALL. Deletion of a single *IKZF1* allele or mutations of a single copy of *IKZF1* were firstly detected in 15 % of all cases of pediatric B-cell ALL and in more than 80 % of Ph+lymphoid leukemia cases, either de novo Ph+ ALL or chronic myeloid leukemia at progression to lymphoid blast crisis [15, 22, 23]. By contrast, *IKZF1* alterations were uncommon in other ALL subtypes that otherwise harbor multiple DNA copy-number alterations, such as *ETV6-RUNX1* ALL, suggesting that *IKZF1* alteration is a key determinant of the lineage and progression of Ph+ leukemia. The analysis of a twin pair concordant for ALL showed that in childhood Ph+ ALL, *BCR-ABL1* gene fusion can be a prenatal and possibly initiating genetic event whereas deletion of *IKZF1* is a secondary and probable postnatal mutation that is associated with poor prognosis [24]. The deletions either involve the entire *IKZF1* locus, resulting in loss of function, or delete an internal subset of *IKZF1* exons, resulting in the expression of dominant negative *IKZF1* alleles. Expression of such dominant negative *IKZF1* alleles in hematopoietic progenitors impairs lymphoid development [25], and loss of *IKZF1* accelerates the onset of Ph+ ALL in a retroviral bone marrow transplant and transgenic models of this disease [26]. Several studies demonstrated that *IKZF1* deletions are significantly associated with an increased relapse rate and adverse events and are correlated with poor outcome in patients with Ph+ ALL [27–29]. Alteration of *IKZF1* is also associated with poor outcome in *BCR-ABL1*-negative ALL [27, 29–31], where this association is independent of commonly used risk stratification features such as age, sex, white cell count, and levels of minimal residual disease (MRD). *IKZF1* deletions and nonsense mutations identified at diagnosis are preserved at relapse [27] and may be used for highly sensitive MRD tests in addition to the repertoire of MRD markers currently available for monitoring MRD in ALL [32]. Importantly, high-risk *BCR-ABL1* negative ALL cases with deletion of *IKZF1* and poor outcome have a gene expression profile significantly similar to that of Ph+ALL (“*BCR-ABL1*-like” ALL) [29, 33], suggesting that these cases may harbor alternative genetic events leading to aberrant activation of tyrosine kinase signaling pathways similar to those downstream of *BCR-ABL1*.

##### PAX5

*PAX5* encodes a transcription factor known as B-cell-specific activator protein, that plays a key role in B-cell commitment by activating essential components of B-cell receptor signaling and repressing the transcription of genes necessary for T-lymphopoiesis [34]. By SNP arrays monoallelic deletion of *PAX5* has been observed in about 30 % of both children and adults with B-ALL, resulting in loss of Pax5 protein expression or in the production of a Pax5 isoform lacking the DNA binding domain and/or transcriptional regulatory domain [5, 17]. Inactivating point mutations in *PAX5* are also observed (7–30 %) as well as chromosomal translocations involving multiple partners such as *ETV6*, *ENL*, *FOXP1, ZNF521, PML, C20ORF112, AUTS2, JAK2, POM121, HIPK1, DACH1, LOC392027, SLCO1B3, ASXL1,* and *KIF3B* [5, 35, 36]. In these rearrangements the DNA binding paired domain of *PAX5* and/or a variable amount of the C-terminal trans-activating domains are fused to functional domains of the partner genes, resulting in a loss of Pax5 function rather than in a gain of functional elements. Alterations of *PAX5* have been demonstrated to not influence treatment outcome [17, 29].

#### Genetic Alterations in Tumor Suppressors Genes

##### CDKN2A/B

The *CDKN2A/B* locus is particularly noteworthy since it encodes for the INK4-class cyclin dependent kinase inhibitors p15^*INK4B*^, p16^*INK4A*^ and for p14^*ARF*^, which inhibits MDM2’s E3 ligase activity stabilizing p53. Alterations have been demonstrated to frequently occur in all lymphoid malignancies, with homozygous deletions as the most frequent mechanism of inactivation [37]. Recently, we identified *CDKN2A* and *CDKN2B* deletion at diagnosis in 29 % and 25 % of *BCR-ABL1-*positive ALL patients, respectively. Deletions were predominantly monoallelic and in more than half of cases the minimal overlapping region of the lost area included a large number of genes. The detection rate of *CDKN2A/ARF* loss increased at relapse compared to diagnosis [18•]. The association with prognosis is still controversial. Some studies reported a correlation of *CDKN2A/B* deletion with poor prognosis, whereas others show no correlation [38]. Confirming previous findings from mice models [39], we demonstrated that deletions of *CDKN2A/B* are significantly associated with higher white blood count and poor outcome in terms of overall survival, disease-free survival, and cumulative incidence of relapse in adult *BCR-ABL1*-positive ALL [18•].

##### Deregulated Expression of CRLF2

Four independent groups in late 2009 and early 2010 have identified that up to 50 % of *BCR-ABL1*-like ALL cases have dysregulated expression of *CRLF2*, the gene encoding the cytokine receptor-like 2 factor, also known as thymic stromal lymphopoietin receptor [40–43]. *CRLF2* has a role in T-lymphoid and dendritic cell development and is involved in inflammation and allergic responses [44]. Although it also mediates B-cell precursor proliferation and survival, its role in B-lymphoid neoplasms needs to be understood [45]. *CRLF2* rearrangements include a translocation of *CRLF2*, which is located at the pseudoautosomal region 1 (*PAR1*) of chromosome Xp/Yp, into the immunoglobulin heavy chain locus at chromosome 14q or a focal *PAR1* deletion proximal of *CRLF2* that results in a novel fusion *P2RY8-CRLF2* [40–43]. Less commonly, a missense mutation in exon 6, F232C, results in constitutive *CRLF2* dimerization [43]. All these events result in overexpression of full-length *CRLF2* on the surface of leukemic cells harboring the genetic alterations, providing a cell-surface marker amenable to detection by flow cytometry for clinical diagnostic purposes. Overall aberrant expression of *CRLF2* was found in 12.5 % to 15 % of B-ALL that lacks typical chromosomal rearrangements, but was not overexpressed in B-ALL cases that have recurring rearrangements or in other lymphoid malignancies [40–43]. *CRLF2* alteration is seen at low rates (5–7 %) when all B-ALL cases are grouped together; however, it is seen in 50–60 % of Down Syndrome (DS) associated ALL, suggesting that *CRLF2* overexpression is especially relevant to tumorigenesis in patients with trisomy 21 [41–43]. In high-risk B-ALL, rearrangements of *CRLF2* are frequently found together with *IKZF1* alterations and activating mutations in *JAK1* and *JAK2*, most commonly at or near R683 in the pseudokinase domain of *JAK2* [40–42]*,* and are associated with very poor outcome [46, 47]. Therapeutically important, *JAK2* mutant-*CRLF2*–mediated and *CRLF2* mutant–mediated transformation are sensitive to *JAK* inhibition in vitro, suggesting that ALL patients with *CRLF2* overexpression may benefit from future kinase inhibitor approaches [41].

##### Intrachromosomal Amplification of Chromosome 21

Intrachromosomal amplification of chromosome 21 (iAMP21) occurs at an incidence up to 2 % in older children with B-cell precursor ALL. iAMP21 was originally identified as a consequence of routine screening for the presence of the *ETV6-RUNX1* fusion by FISH [48] and is defined by at least three copies of the *RUNX1* gene, commonly concomitant with deletion of the subtelomeric regions of chromosome 21. Recently, the common region of amplification on chromosome 21 was better refined to a 5.1-Mb region that included *RUNX1*, miR-802, and genes mapping to the DS critical region. Recurrent abnormalities affecting *IKZF1* (22 %), *CDKN2A/B* (17 %), *PAX5* (8 %), *ETV6* (19 %), and *RB1* (37 %) and secondary to chromosome 21 rearrangements were identified [49]. iAMP21 has been shown to be linked to a dismal outcome when patients are treated with standard therapy, because it is associated with an increased risk of both early and late relapses [50, 51].

### Prognostic Factors Based on Conventional and Next-Generation Sequencing Technologies

Whole genome/exome-sequencing approaches have been demonstrated to provide a comprehensive view of the landscape of genetic alterations in leukemia identifying crucial markers for diagnosis, risk-stratification, and potentially novel target therapies. Screening for *BCR-ABL1* mutations in Philadelphia-positive ALL allows to identify patients who may benefit from second-generation TKIs or from novel compounds targeting the T315I mutation [52]. An extensive Sanger resequencing of 120 candidate cancer genes in diagnostic leukemia samples from 187 children and adolescents with high-risk B-ALL treated with augmented post-induction chemotherapy on the Children’s Oncology Group P9906 protocol and integrated analysis with genome-wide CNAs and gene expression profiles revealed recurrent somatic alterations in key signaling pathways, including B-cell development/differentiation (68 %), *TP53/RB* tumor suppressor pathway (54 %), Ras signaling (50 %), and Janus kinases (11 %). The frequency of mutations within the four major pathways varied markedly across genetic subtypes with a striking association of B-cell development/differentiation and *JAK2* mutations with leukemias expressing a *BCR-ABL1*-like gene expression profile and of Ras signaling pathway mutations with cases defined by a distinct gene expression profile coupled with focal *ERG* deletion [53]. For most of these genes the prognostic impact remains to be determined, but they highlighted important new therapeutic targets for selected patient subsets. The landscape of genetic alterations in B-ALL was extended by the identification of loss-of-function mutations (either deletions or sequence mutations) of *CREBBP*, encoding the transcriptional coactivator and histone acetyltransferase CREB-binding protein or CBP, in 19 % of relapsed samples from children with B-cell progenitor ALL [54•]. Functionally, the mutations impair histone acetylation and transcriptional regulation of *CREBBP* targets, including glucocorticoid responsive genes, likely influencing treatment responsiveness in ALL. *CREBBP* mutations are preserved at relapse when present at diagnosis, acquired at relapse but occurring in minor subclones at diagnosis, or reduplicated to homozygosity at relapse when heterozygous at diagnosis [54•]. Although the prognostic impact remains to be determined, the observation that the *CREBBP* mutations impair regulation of glucocorticoid-responsive genes, and that the mutations are selected for at relapse, suggests that these alterations may influence the likelihood of relapse. Therefore, novel therapeutic approaches, such as histone deacetylase inhibitors (HDAC) [55], directed at modulating protein acetylation may be useful in high-risk ALL. Recently, new molecular prognostic markers have been identified. The analysis of 265 first-relapse patients enrolled in the German Acute Lymphoblastic Leukemia Relapse Berlin-Frankfurt-Münster 2002 (ALL-REZ BFM 2002) trial for sequence and copy number alterations of the *TP53* gene, by using direct sequencing and multiplex ligation-dependent probe amplification, identified copy number and sequence alterations of *TP53* in 12.4 % of B-cell precursor ALL samples [56]. In more than half of cases (54 %) *TP53* alterations were gained at relapse. Mutations were strikingly associated with non-response to chemotherapy and poor event-free survival and overall survival rates, allowing the identification of patients at high risk of treatment failure. To overcome the limits of conventional Sanger sequencing whole exome and transcriptome NGS approaches have been subsequently pursued in two pediatric *ETV6-RUNX1* (*TEL-AML1*) positive ALL [57] and in an adult patient with *BCR-ABL1* positive ALL [58], respectively. Both studies identified novel somatic mutations, some of them occurred in genes previously described to be mutated in other types of cancer, but none was found to be recurrent in an extended series of ALL samples. Although the true pathogenetic significance of the mutations must await future functional evaluations, these studies provided a first estimate of the mutational burden of *ETV6-RUNX1* and *BCR-ABL1*-positive ALLs.

## T-Progenitor Acute Lymphoblastic Leukemia

### Prognostic Factors Based on Cytogenetics

T-ALL is a malignant disorder of T-cell lymphoid progenitor cells that affects 15 % of children and 25 % of adults with ALL. Structural chromosomal aberrations are identified in approximately 50 % of cases and frequently involve the juxtaposition of strong promoter and enhancer elements from T-cell receptor (TCR) genes with transcription factor genes as consequence of an illegitimate event during V(D)J recombination in normal T-cell development. This leads to the aberrant expression of the fusion partners resulting in thymocytes differentiation block at various stages of maturation.

#### TCR Gene Rearrangements

The most common rearrangements include TCR alpha/delta chain at 14q11.2, TCR beta chain at 7q34, and TCR gamma chain at 7p14. With few exceptions, the involved gene on the partner chromosome encodes a cell cycle inhibitor or a transcription factor whose expression is deregulated or activated as a result of the rearrangement. *TAL1* (1p32) is ectopically expressed in T-ALL as consequence of t(1;14)(p32;q11) [59] (3 % in childhood T-ALL) and more frequently as a consequence of the intrachromosomal deletion resulting in *SIL-TAL1* fusion gene, while *LYL1* (19p13)*, TAL2* (9q32), and *BHLH1* (21q22) are up-regulated in the rare translocations t(7;19)(q34;p13), t(7;9)(q34;q32), and t(14;21)(q11;q22), respectively. Their aberrant expression may contribute to leukemia through the formation of heterodimers with class I basic helix-loop-helix members that regulate T-cell specific genes, with consequent differentiation and proliferation impairment. LIM domain only genes *LMO1* (11p15) and *LMO2* (11p13) are involved in t(11;14)(p15;q11) and t(11;14)(p13;q11) with *TCR* alpha/delta loci and in translocations with *TCR* beta. LMO abnormal expression associates with deregulation of *LYL1* and *TAL1*, even in absence of specific translocations [60]. The homeobox (HOX) genes are a highly conserved family of transcription factors that play an important role in morphogenesis during embryonic development and in normal hemopoiesis [61]. The inv(7)(p15q34) and t(7;7)(p15;q34) bring the *TCR* beta regulatory elements in the vicinity of the *HOXA* genes cluster disrupting the normal regulatory elements of the cluster with subsequent up-regulation, especially of *HOXA9, HOXA10,* and *HOXA11* [62, 63]. *TLX1* (*HOX11*) is expressed at high level in more than 30 % of adult T-ALL as consequence of t(10;14)(q24;q11) and t(7;10)(q34;q24), while *TLX3* (*HOX11L2*) is involved in t(5;14)(q35;q32) with the fusion partner *BCL11B* in about 20 % of children and 4 % of adults [64]. Additional genes rarely involved in *TCR* loci rearrangements are *LCK* [65], *CCND2* [66], and *IRS4* [67].

#### Non-TCR Loci

A variety of cytogenetic abnormalities can occur in T-cell ALL that do not involve the TCR loci. These include del(6q), t(11q23) (MLL gene), t(14q32), trisomy 8, and t(10;11). A favorable prognostic correlation has been assessed for t(10;14), which is extremely rare in adult patients, and more frequent in the pediatric subset. Trisomy of chromosome 8 and monosomy of chromosome 7 usually carry a bad prognosis [68]. The cryptic deletion del(9)(q34.11q34.13) results in the *SET-NUP214* fusion product, which transcriptionally activates specific members of the *HOXA* cluster maybe contributing to T-ALL pathogenesis by the inhibition of T-cell maturation [69]. The *ABL1* cytoplasmic tyrosine kinase plays a role in T-cell signaling, leading to the induction of IL-2 production and proliferation following TCR activation [70]. *ABL1* fusion genes can be identified in about 8 % of T-ALL. *EML1-ABL1* fusion due to a cryptic t(9;14)(q34;q32), *BCR-ABL1* t(9;22)(q34;q11), and *ETV6-ABL1* t(9;12)(q34;p13) are seldom reported chimeric genes in T-ALL, although if frequent in other hematologic malignancies. In contrast, the most frequent and strictly associated alteration with T-ALL is the *NUP214-ABL1* fusion identified in 6 % of cases, in both children and adults, also if it is not clear its prognostic relevance [71]. *JAK2* resulted constitutively activated in the rare t(9;12)(p24;p13) encoding *ETV6-JAK2* fusion oncoprotein, while *JAK1* is widely mutated in adult T-ALL (18 %) and less in children (3 %), and associates with a poor response to therapy [72]. Finally, a recent study identified a novel fusion partner of *ETV6* in one T-ALL patient, harboring *ETV6-ARNT* t(1;12)(q21;p13) [73].

### Prognostic Factors Based on Genomic Profiling

On the basis of gene expression profiling, T-ALL cases can be classified into major subgroups that are indicative of leukemic arrest at specific stages of normal thymocyte development (*HOX11* ^+^ early cortical thymocytes; *LYL1* ^+^ early pro-T thymocytes; *TAL1* ^+^ late cortical thymocytes) and have clinical relevance, because they are associated with a favorable or worse prognosis [60]. Using SNP array platforms, many novel genomic alterations have recently been identified, including focal deletions of *RB1* [5], duplications of the proto-oncogene *MYB* [5, 74], deletion of 9p21.2 in more than 70 % of patients [75], deletion and mutation of *PTEN* [5, 76], and deletion or mutation of the U3 ubiquitin ligase *FBXW7* [77, 78]. Thus far, mutations of *NOTCH1* and *FBXW7* have generally been associated with a favorable prognosis [79]. *NOCTH1* role in leukemogenesis was initially identified in the rare chromosomal translocation t(7;9)(q34;q34.3) that fuses the intracellular form of *NOTCH1* to the TCR beta and leads to the expression of *TAN1*, a truncated and constitutively activated form of *NOTCH1*; subsequently, activating or loss of function mutations has been identified in more than 50 % of T-ALL cases, representing the most common alteration in T-ALL. The high frequency of *NOTCH1* mutations in T-ALL has sparked an interest in the development of anti-*NOTCH1* targeted therapies for the treatment of T-ALL. Other mutated genes in T-ALL are *WT1*, *NRAS*, the negative regulator of the RAS pathway, *NF1* that is inactivated because of deletions or mutations [80], and rarely *FLT3* [81, 82] and *PTPN2* [83], that are affected by activating mutations and focal deletions, respectively. All these genes play crucial roles as regulators and alterations in their function may affect critically different signal transduction pathways. Of note, identification of 6q15-16.1 deletion containing *CASP8AP2* gene represents a novel prognostic factor that defines a high-risk group (Table 2) [84].

**Table 2:** Novel recurring genetic alterations occurring in T-progenitor ALL and their correlation with outcome

Gene name	Alteration	Frequency	Prognosis	Reference
*NOTCH1*	Sequence mutations	~50 % of T-ALL	Associated with favorable outcome in children	[89–92]
*FBXW7*	Sequence mutations	~20 % of T-ALL	Associated with favorable outcome in children	[78, 91, 92]
*PTEN*	Deletions or sequence mutations	6–8 % of T-ALL	Associated with poor response to chemotherapy and resistance to pharmacological inhibition of *NOTCH1*	[76, 93]
*CDKN2A/B*	Deletions	30–70 % of T-ALL	Associated with poor outcome in adult and children T-ALL	[94, 95]
*CDKN1A*	Deletions or sequence mutations	12 % of T-ALL	To be investigated	[84, 96]
6q15-16.1	Deletion	12 % of T-ALL	Associated with poor outcome	[84]
*PHF6*	Deletions or sequence mutations	16 % of pediatric T-ALL cases; 38 % of adult T-ALL cases	Associated with reduced overall survival	[85]
*WT1*	Frameshift mutations	13 % of pediatric T-ALL cases; 12 % of adult T-ALL cases	No association with outcome	[97, 98]
*LEF1*	Focal deletions or sequence mutations	15 % of pediatric T-ALL cases	Associated with younger age and a trend toward a better overall survival	[99]
*JAK1*	Sequence mutations	18 % of adult T-ALL cases	Associated with reduced disease-free survival and overall survival	[72]
*FLT3*	Internal tandem duplication	4 % of adult T-ALL cases; 3 % of pediatric T-ALL cases	No association with outcome	[81, 82]
*PTPN2*	Deletion	6 % of T-ALL	Down-regulation of *PTPN2* expression results in prolonged survival of ALL-SIL cells after imatinib treatment	[100]

### Prognostic Factors Based on Next-Generation Sequencing Technology

Using exon capture of chromosome X a recent study by Van Vlierberghe and colleagues [85] identified inactivating mutations of the X-linked plant homeodomain finger 6 (*PHF6*) in 16 % of pediatric and 38 % of adult T-ALL cases. *PHF6* mutations were almost exclusively found in male and were associated with leukemias driven by aberrant expression of *TLX1* and *TLX3*. Although the prognostic significance remains to be determined, *PHF6* has emerged as a new X-linked tumor suppressor in T-ALL. Recently, the application of whole-genome sequencing (WGS) to the characterization of “early T-cell precursor” (ETP) ALL, that comprises up to 15 % of T-ALL and is associated with a high risk of treatment failure, has provided potential new molecular targets for therapy [86••]. Zhang and colleagues [86••] performed WGS of 12 ETP ALL cases identifying activating mutations in genes regulating cytokine receptor and RAS signaling, such as *NRAS, KRAS, FLT3, IL7R, JAK3, JAK1, SH2B3,* and *BRAF* (67 %), inactivating lesions disrupting hematopoietic development, such as *GATA3, ETV6, RUNX1, IKZF1,* and *EP300* (58 %), and histone-modifying genes, such as *EZH2, EED, SUZ12*, *SETD2,* and *EP300* (48 %). Moreover, they identified new targets of recurrent mutation in *DNM2, ECT2L,* and *RELN* genes. Importantly, the gene expression profile of ETP ALL resulted similar to that of normal and myeloid leukemia hematopoietic stem cells, suggesting the possibility that myeloid-directed therapies might improve the poor outcome of ETP ALL.

## Conclusions

Thanks to the use of SNP microarray-based technology, extensive candidate gene sequencing, and whole genome/exome/transcriptome NGS approaches, novel and recurring lesions have been identified in B and T-ALL. The identification of these alterations allowed a better classification of leukemia patients in subgroups according to specific genetic lesions (eg, *BCR-ABL1* like subgroup), provided new markers for risk-assessment and/or for monitoring minimal residual disease (eg, *IKZF1*, *CRLF2*, *TP53*), and highlighted novel therapeutic approaches (eg, inhibitors of *JAK2*, *HDAC,* and *NOTCH1*). However, much remains to be investigated. From the biological prospective, whole exome/transcriptome NGS approaches will provide a comprehensive view of the complexity of genetic alterations in ALL, identifying genes and pathways relevant for the establishment of the leukemia clone and/or for the disease recurrence and responsible for responsiveness to therapy. From the clinical prospective, all these findings should be integrated into the clinic, providing new prognostic markers and novel targets for tailored therapies.
